# Variable temperature solid-state NMR spectral and relaxation analyses of the impregnation of polyethylene glycol (PEG) into coniferous wood†

**DOI:** 10.1039/c9ra01848d

**Published:** 2019-05-20

**Authors:** Masakazu Nishida, Tomoko Tanaka, Tsunehisa Miki, Ichinori Shigematsu, Kozo Kanayama

**Affiliations:** National Institute of Advanced Industrial Science and Technology (AIST) 2266-98 Shimoshidami, Moriyama-ku Nagoya 463-8560 Japan m-nishida@aist.go.jp +81 52 736 7403 +81 52 736 7493; Research Institute for Sustainable Humanosphere, Kyoto University Gokanosho Uji Kyoto 611-0011 Japan

## Abstract

To investigate the behaviours of polyethylene glycol (PEG) and its interaction with biomass constituents in coniferous wood (Japanese cypress), variable temperature solid-state NMR spectra and relaxation times were measured from 20–80 °C. Signal intensities in the ^1^H and ^13^C PST-MAS NMR spectra changed depending on both the measurement temperature and the melting point of the impregnated PEG. In the ^13^C CP-MAS NMR spectra with increasing temperature, although the signal intensities of biomass constituents slightly decreased, signal intensities of PEG molecules in the cypress maximized at 80 °C. PEG impregnation into cypress decreased the *T*_1_H values at 80 °C for short to medium chain PEG in the liquid phase while it decreased *T*_1_H values at ambient temperature for long chain PEG in the solid phase because the interactions of PEG molecules and the biomass constituents of coniferous wood were different for different chain lengths of the PEG. These variable temperature measurements of both solid-state NMR spectra and relaxation time indicated that impregnation of longer chain PEG molecules produced higher hydrophobicity because of the increased steric hinderance of PEG attached to carbohydrates. The variable temperature measurements also showed that long chain PEG molecules were restricted to the lumen while short to medium chain length PEG molecules infiltrated into the intercellular region of the cell wall in addition to the lumen. These results obtained from the variable temperature NMR measurements were also supported by ATR-IR spectroscopy analyses.

## Abstract 2

To investigate the behaviours of polyethylene glycol (PEG) and its interaction with biomass constituents in coniferous wood (Japanese cypress), variable temperature solid-state NMR spectra and relaxation times were measured from 20–80 °C.

## Graphical abstract

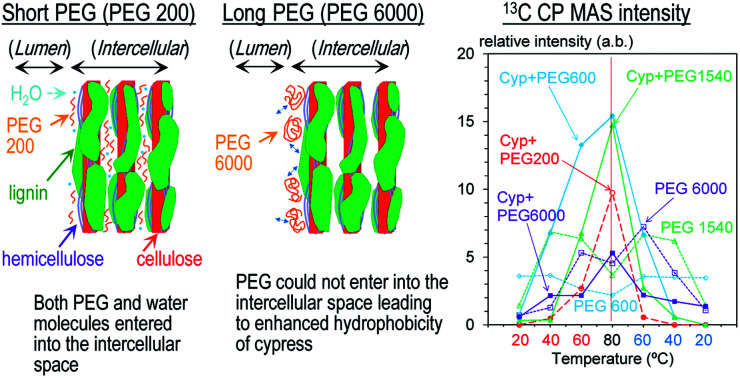

## Introduction

A wide spectrum of research is being carried out on biomass resources because they contribute to a recycling-oriented society from the viewpoint of carbon fixation, in addition to the substitution of petroleum resources. With respect to the industrial materialization of woody bioresources, chemical modifications are most useful, especially for fabrication processes utilizing solid wood mass as well as for the use of individual biomass constituents in the solid wood as engineering resources.^1^ Polyethylene glycol (PEG) is a chemical of choice for modifying woody materials thanks to its easy handling and low toxicity. Because of its hydrophilicity and non-reactivity, PEG has been used as a stabilizing reagent for various types of woody materials. In particular, dimensional control of wood with PEG had been a well-known method for a long time^2–4^ and is established as one of the significant chemical processing methods for wood.^5^ Very recently, dimensional stabilization of wood was studied for a variety of PEG impregnations with energy-dispersive X-ray spectroscopy (EDX) area-measurements.^6^ Since PEG impregnation is easy to use outside a laboratory, PEG treatments have been widely applied in the conservation of waterlogged archaeological wood.^7^ With respect to applications in wood preservation, a formulation of tannin-PEG was also to study weathering properties using Fourier-transform infrared (FT-IR) and ^13^C solution nuclear magnetic resonance (NMR) spectroscopies.^8^ PEG was also used as a grafting reagent to stabilize the dimensions of a cellulose microcrystal suspension.^9^

Furthermore, the manufacture of wood polymer composites (WPC) is an important industrial topic. Some recent studies of biomass-based composites containing PEG, for application in various fields have been published. A wood composite impregnated with PEG after ultrasonic treatment showed high compressive strength because PEG has a low friction coefficient.^10^ In an application to multi-component biomass-based composites, PEG was used as a plasticizer for a poly(3-hydroxybutyrate) (PHB)/beech wood flour composite.^11^ For wood–hydroxyapatite composite materials, PEG was used as a grafting agent on the surface of wood, providing a uniform and microporous morphology.^12^ Film-shape composites consisting of natural wood and ionic liquids were prepared together with PEG, chitosan, and multi-wall carbon nanotubes.^13^ For wound dressing applications, nanocellulose films were manufactured with PEG and were evaluated by mechanical and printing tests.^14^ Electron-beam irradiation polymerized acrylate-functional PEG oligomers made woody materials more durable and less sensitive to weathering.^15^

In addition to the uses of PEG in product manufacture described above, PEG can be used for manufacturing raw materials for industry. As an example of the PEG solvolysis product, PEG-modified glycol lignin was isolated and its thermal properties were examined by thermomechanical analysis (TMA) and thermogravimetric analysis (TGA).^16^ The potential of PEG has been extended to an application involving an enzymatic reaction of woody materials. The pre-treatment of softwood with PEG was effective for enzymatic saccharification at higher substrate concentrations.^17^ Medium molecular weights of PEG (*M*_w_ 400, 600, 1000) were also applied to the liquefaction of enzymatic hydrolysis lignin.^18^

In summary, the use of PEG in manufacturing processes covers a wide range of fields in applications involving woody materials and the mechanical and thermodynamic properties of both raw materials and products have been extensively studied. Meanwhile, several studies have been published about the behaviour of PEG with reference to manufacturing at the molecular to nano scales. Light-induced changes in the surface of PEG-impregnated wood were examined by FT-IR photoacoustic spectroscopy (PAS).^19^ Matrix-assisted laser desorption/ionization time-of-flight (MALDI-TOF) measurements showed molecular weight distributions in wood cell walls.^20^ However, these methods could not provide information about the many interactions of PEG molecules and biomass constituents in woody materials. Moreover, solid-state NMR is known as a useful analytical method for wood and woody products.^21^ As an application of solid-state NMR to the PEG impregnation process, archaeological wood was evaluated with ^13^C cross-polarization and magic-angle spinning (CP-MAS) NMR.^22^ Although ^13^C CP-MAS NMR proved to be a useful method for the investigation of archaeological woods, it was difficult to assess the behaviour of PEG molecules by this method using measurements at ambient temperature alone.

We have been investigating the use of analytical methods, using mainly solid-state NMR, intended for the development of quality control in manufacturing processes for natural biomass resources. As solid-state NMR uses low energy radio waves, it is a non-destructive analytical method. Moreover, since the radio waves permeate into the materials, solid-state NMR is also suitable for bulk analysis without special pre-treatments. Therefore, solid-state NMR can monitor the manufacturing process of biomass resources having a complex structure inside in the natural state. From the viewpoint of hierarchal spatial levels of biomass resources, we have accomplished multi-scale instrumental analyses using solid-state NMR for molecular to nano scale evaluations as well as using electron microscopy or X-ray computed tomography (CT) for micro scale evaluation. This multi-scale approach was used to study the nanostructures of plant materials^23,24^ as well as the compatibility of biomass-based polymer blends.^25^ Meanwhile, concerning the molecular mobility of biomass constituents in woody materials, we also investigated the use of integrated analysis of solid-state NMR spectra and nuclear magnetic relaxation times for studying chemical modifications of cypress.^26^

In the present study, the behaviours of various sizes of PEG molecules and their interactions with biomass constituents were examined by analysing correlations of data from several variable-temperature solid-state NMR methods, based on the knowledge obtained from previous multi-scale instrumental analyses and integrated NMR analyses. In solid-state NMR, the measurement temperature can be easily changed without any special apparatus. Therefore, variable-temperature solid-state NMR as the non-destructive bulk analysis can evaluate changes of behaviours and interactions of both PEG molecules and biomass constituents during a phase transition near the melting point of PEG. In particular, we focused on coniferous wood (Japanese cypress) impregnated with low (*M*_w_ 200) to high (*M*_w_ 6000) molecular weight PEG, using not only three type of solid-state NMR spectra but also relaxation time measurements for a cycle of rising and dropping temperatures over the range from 20 to 80 °C. Water molecules were evaluated with ^1^H MAS NMR spectra while biomass constituents in cypress were evaluated with ^13^C CP-MAS NMR spectra. Furthermore, PEG molecules in the impregnated cypress were mainly evaluated with ^13^C pulse transfer saturation and magic-angle spinning (PST-MAS) in addition to ^1^H MAS NMR spectra. Combining these with the ^1^H spin-lattice relaxation time in the laboratory frame, the responses of each solid-state NMR spectra to the cycle of temperature were analysed in order to obtain information about behaviours and molecular interaction of PEG molecules in the impregnated cypress. We also confirmed the solid-state NMR results by using attenuated total reflection infrared (ATR-IR) as an alternative method to investigate the functional groups in the impregnated cypress.

## Experimental

### Materials

From a sapwood of Japanese cypress (Hinoki) (*Chamaecyparis obtusa*), consecutive 3.5 mm thick discotic plates with a 40 mm diameter were cut for matching fibre directions using a NC milling machine. The cut cypress plate was heat-dried at 105 °C for 2 h (fully-dried bulk density: 0.39 g cm^−3^) and then was stored in a desiccator to 8% moisture content (the reference cypress). Various molecular weight fractions of polyethylene glycol (PEG) were purchased from Wako Pure Chemical Co. Ltd (Osaka, Japan). The species of PEG used for impregnation together with their melting points were as follows: PEG 200 (*M*_w_ 180–220, mp −65 to −50 °C); PEG 600 (*M*_w_ 560–640, mp 15–25 °C); PEG 1540 (*M*_w_ 1500, mp 40–50 °C); PEG 6000 (*M*_w_ 6000, mp 50–65 °C). The reference cypress was impregnated with a 15% aqueous solution of each PEG under vacuum at ambient temperature. After the PEG-impregnated cypress was dried in air for 48 h, using a drying machine at 35 °C, the dried PEG-impregnated cypress was further dried with an air flow for 96 h and under vacuum for 24 h. The weight percent gain (WPG) of the dried PEG-impregnated cypresses fell within the range of 24–27%. The reference sample and the PEG-impregnated cypress were obtained from the same plate cut along the same fibre directions. The NMR spectrum and relaxation time measurements used the PEG-impregnated cypress stored in desiccator at 20 °C and 30% relative humidity for 24 h.

### Solid-state NMR measurements

Magic angle spinning (MAS) nuclear magnetic resonance (NMR) spectra were measured on a Varian 400 NMR system spectrometer (Palo Alto, CA) with a Varian 4 mm double-resonance T3 solid probe. The samples stored in a desiccator were transferred to a 4 mm ZrO_2_ rotor spun at 15 kHz. For variable temperature measurements, the temperature of the sample inlet was carefully controlled using a dry nitrogen flow over a temperature range of 20 to 80 °C, unless otherwise noted. ^1^H MAS NMR spectra were collected with a 2.9 μs π/2 pulse at 399.86 MHz for the ^1^H nuclei using a 40 ms acquisition period over a 30.5 kHz spectral width in 16 transients, and a 3 s recycle delay. ^13^C MAS NMR spectra were collected with 2.6 μs π/2 pulse at 100.56 MHz for the ^13^C nuclei and a 40 ms acquisition period over a 30.7 kHz spectral width. Proton decoupling was performed with an 86 kHz ^1^H decoupling radio frequency with a small phase incremental alteration (SPINAL) decoupling pulse sequence.^27^ Cross-polarization/magic angle spinning (CP-MAS) NMR spectra were measured with a 5.0 s recycle and 1024 transients delay, using a ramped-amplitude pulse sequence^28^ with a 2 ms contact time and a 2.5 μs π/2 pulse for the ^1^H nuclei. The amplitude of the ^1^H pulse was ramped up linearly from 90.5% of its final value during the cross polarization contact time. The contact time was optimized by measurements of several selected samples with variable contact times (100–8000 μs). Pulse saturation transfer/magic angle spinning (PST-MAS) NMR was measured using a single π/2 pulse for the ^13^C nuclei with a 3 s recycle delay in 2048 transients after saturation of ^1^H nuclei with 14 consecutive 2.5 μs pulses and a 27.5 μs delay. The ^1^H spin-lattice relaxation time in the laboratory frame (*T*_1_H) was directly measured by the saturation recovery method with 13 consecutive 2.5 μs pulses and a 27.5 μs delay for the ^1^H nuclei. The *T*_1_H analyses were performed with the same solid-state probe and conditions used for the ^1^H MAS NMR spectrum.

### Infrared spectroscopy

Fourier transfer infrared (FT-IR) spectra were measured on a Thermo Scientific Nicolet 6700 FT-IR spectrometer (Waltham, MA) with 4 cm^−1^ resolution and 32 scans in the range 700–4000 cm^−1^. Attenuated total reflection (ATR) data was collected using a Thermo Scientific SMART iTR single reflection diamond ATR attachment (Waltham, MA) at ambient temperature.

## Results and discussion

### Variable temperature ^1^H MAS NMR spectral changes of PEG-impregnated Japanese cypresses

The ^1^H signal line shape in ^1^H MAS NMR depends on the molecular motions involved in *T*_2_ relaxation, and has been used for biodegradable polymer.^25^ As a part of the study of the effects of various molecular weight polyethylene glycols (PEGs) on solid-state NMR spectra of cypress, state changes around the melting point were investigated by ^1^H MAS NMR spectral analysis. In addition, the interactions of each type of PEG molecule with biomass constituents were examined by comparison of ^1^H MAS NMR spectra between the PEG-impregnated cypress and PEG molecules in pure form.

**Fig. 1:**
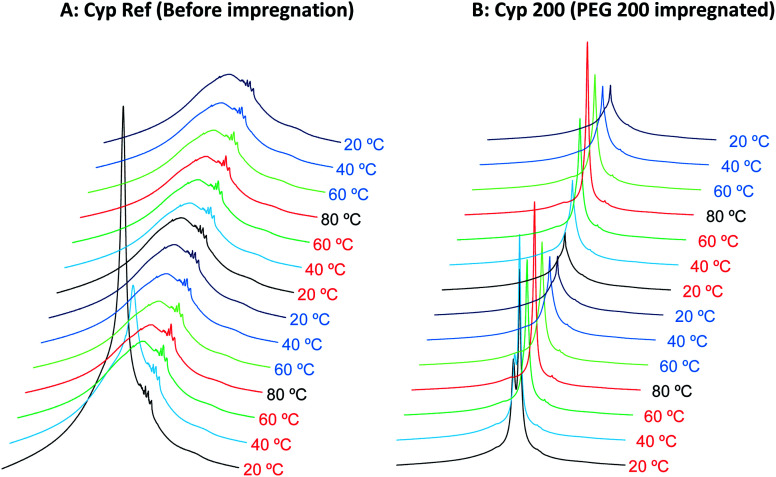
^1^H MAS NMR spectral changes of Japanese cypress with PEG impregnation for two cycles of rising and lowering temperatures.

First, the cypress impregnated with PEG 200 (labelled as Cyp 200) was compared with the reference sample before the PEG impregnation (labelled as Cyp Ref), as shown in Fig. 1. Even after being in the low humidity in the desiccator, the Cyp Ref sample retained a large amount of water, appearing at 5 ppm. This molecular water signal immediately decreased at 40 °C and it disappeared, leaving the remaining biomass constituents of Cyp Ref over 60 °C. The remaining signals of the Cyp Ref sample stayed unchanged over 60 °C and during the temperature reduction process, and they were unchanged during the second cycle of temperature rise and drop. While the molecular water signal also appeared in the sample Cyp 200, a larger and sharper signal of PEG was observed at higher magnetic field (4 ppm). In the sample Cyp 200, the water signal immediately decreased at 40 °C and disappeared at temperatures over 60 °C. In contrast, the PEG signal maximized at 80 °C and decreased with decreasing temperature for Cyp 200. During the second temperature cycle, the PEG signal increased with increasing temperature and decreased with decreasing temperature, producing a similar signal intensity–temperature curve to the first drop.

**Fig. 2:**
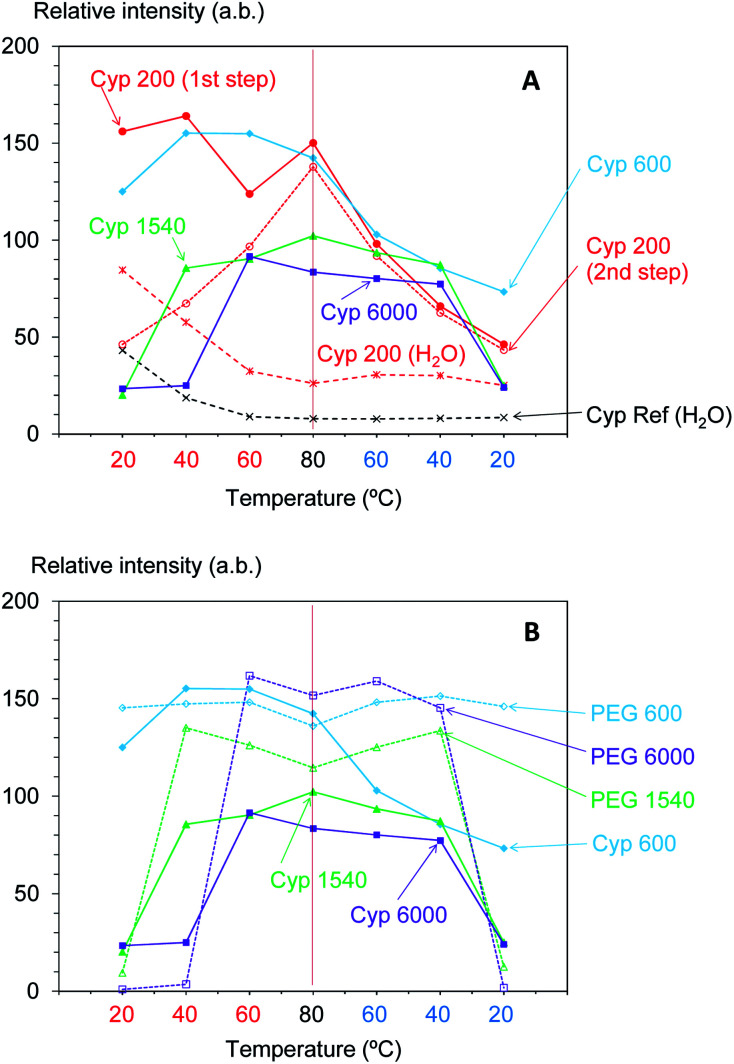
^1^H MAS signal changes of PEG-impregnated Japanese cypresses: (A) comparison of molecular weights of PEG; (B) comparison with PEG in pure form.

Next, the signal intensity changes occurring during in these temperature cycles were examined for various molecular weights of PEG impregnating into cypress. The original ^1^H MAS NMR spectra of PEG-impregnated cypresses at various temperatures are presented in Fig. S1 (ESI).† For each PEG-impregnated cypress, the ^1^H signal intensity changes are summarized in Fig. 2A. As shown in Fig. S1,† cypress impregnated with PEG 600 (labelled as Cyp 600) had less water before the temperature was increased than Cyp 200. The signal intensity of Cyp 600 increased at 40 °C, and then monotonically decreased during the temperature cycle. The divergence of ^1^H signal intensity between the rising and dropping processes was also observed not only for Cyp 600 but also for the first step of Cyp 200 (Fig. 2A). Since water molecules were lost from the PEG-impregnated cypress as the temperature rose, the existence of water molecules caused this divergence.

For cypresses impregnated with larger molecular weight PEG [labelled as Cyp 1540 (PEG 1540) and Cyp 6000 (PEG 6000), respectively], no signals of water molecules were observed in ^1^H MAS NMR measurements even before a temperature rise (Fig. S1†). The ^1^H signal intensity of Cyp 1540 greatly increased after 40 °C in the rising process while it reversibly decreased at 20 °C in the dropping process. Namely, the ^1^H signal of Cyp 1540 showed the same type of intensity–temperature curve in both legs of the temperature cycle (Fig. 2A). Although Cyp 6000 started to afford significant ^1^H signals at 60 °C as the temperature rose, its ^1^H signals still remained at 40 °C as the temperature dropped, producing asymmetrical intensity–temperature curves.

In order to evaluate ^1^H signal changes due to interactions between PEG and biomass constituents of cypress, ^1^H MAS spectra of PEG molecules in pure form were measured. The original ^1^H MAS NMR spectra of selected PEGs (PEG 600, PEG 1540, and PEG 6000) at various temperatures are presented in Fig. S2 (ESI).† The comparison of ^1^H signal intensities between the impregnated cypresses and PEGs is summarized in Fig. 2B. Unlike the Cyp 600 sample which showed a smaller ^1^H signal intensity at 20 °C as the temperature was lowered, the ^1^H signal of PEG 600 slightly decreased with increasing temperature and took a minimum value at 80 °C. With respect to PEG 1540 and PEG 6000, however, the ^1^H signal enlarged near the melting point of both PEGs (60 °C) during rising temperatures while the ^1^H signal was still apparent at temperatures below the melting point (40 °C) as the sample cooled, behaviour also seen for Cyp 1540 and Cyp 6000. However, with decreasing temperature, the ^1^H signals of PEG 1540 and PEG 6000 increased while those of Cyp 1540 and Cyp 6000 decreased. Furthermore, PEG molecules in pure form showed narrower ^1^H signals than corresponding PEG molecules in the impregnated cypress. As well as the ^1^H signal increase depending on the measuring temperature, the broadened ^1^H signals due to the impregnation were also caused by the interaction between PEG and biomass constituents in cypress.

### Variable temperature ^13^C CP-MAS NMR spectral changes of PEG-impregnated Japanese cypresses

We have already studied the crystallinity and molecular mobility of biomass-based polymers using a combination of ^13^C CP-MAS NMR and ^13^C PST-MAS NMR methods.^25^ The former is commonly used method for biomass-based polymers as well as petroleum-derived polymers; it enhances the signals from rigid components with low mobility *via*^1^H–^13^C magnetization transfer. Meanwhile, the ^13^C PST-MAS method uses the nuclear Overhauser effect (NOE) to enhance signals of flexible portions near hydrogen atoms. In order to evaluate the impregnation effect for both biomass constituents and PEG in the impregnated cypress, variable temperature ^13^C CP-MAS and PST-MAS NMR spectra were compared between the cypress before impregnation (Cyp Ref) and the cypress impregnated with PEG 200 (Cyp 200).

**Fig. 3:**
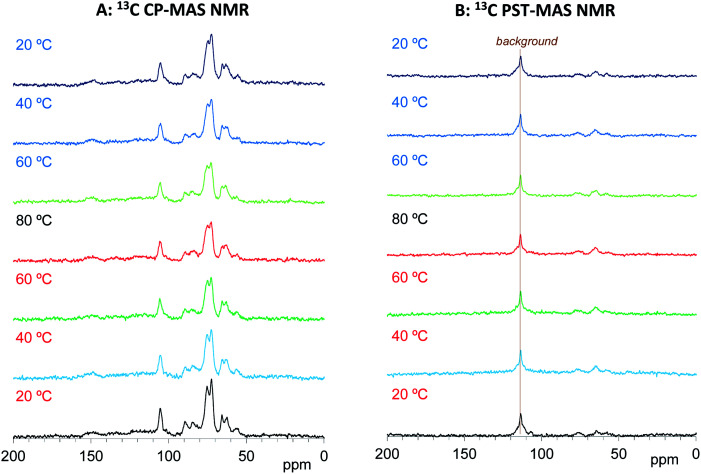
^13^C MAS NMR spectral changes of Japanese cypress before PEG impregnation (Cyp Ref) for a temperature cycle.

As shown in Fig. 3A, the ^13^C CP-MAS NMR spectra of the Cyp Ref sample showed characteristic carbohydrate signals (60–110 ppm) and lignin signals (OCH_3_: 55 ppm; aromatic: 110–160 ppm) before the variable temperature measurement. With increasing temperature, the carbohydrate signals gradually decreased and broadened. These carbohydrate signals increased and sharpened again on cooling, and the ^13^C CP-MAS signals after the variable temperature measurements were smaller compared with those before heating. The reduction and widening of ^13^C CP-MAS signals was caused by the release of water molecules from cypress which reduced the enhancement of the efficiency of the ^1^H–^13^C magnetization transfer. As shown in Fig. 3B, the ^13^C PST-MAS NMR spectra of the Cyp Ref sample gave very small signals, two for carbohydrate (65 and 75 ppm) and one for lignin OCH_3_ (57 ppm), as previously reported for Japanese cypress.^23,26^ These three ^13^C PST-MAS signals slightly increased with increasing temperature and inversely decreased slightly with decreasing temperature. The slight enlargement of ^13^C PST-MAS signals was caused by the increase of molecular mobility due to the temperature rise.

**Fig. 4:**
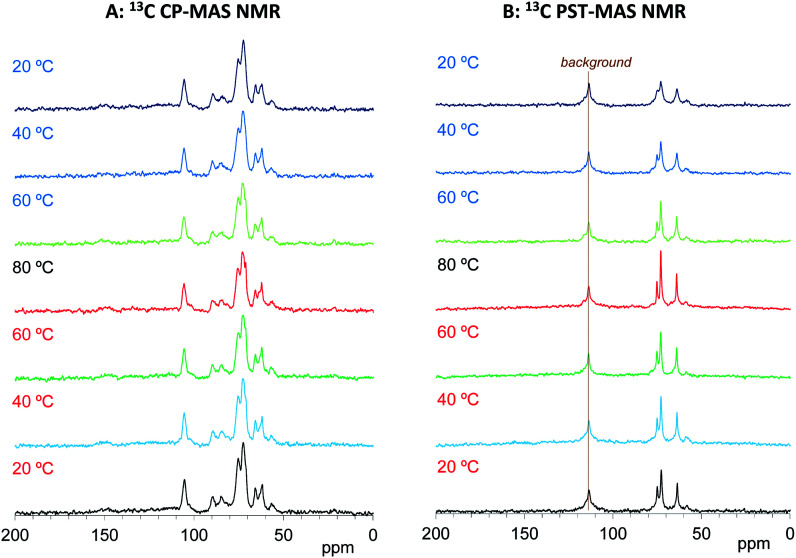
^13^C MAS NMR spectral changes of Japanese cypress impregnated with PEG 200 (Cyp 200) for a temperature cycle.

Based on the signals in the ^13^C CP- and PST-MAS NMR spectra for the Cyp sample Ref, the effects of impregnation on both types of ^13^C MAS NMR spectra were examined. As shown in Fig. 4A, the ^13^C CP-MAS signals of carbohydrates were enlarged by the impregnation of PEG 200. With increasing measurement temperature, these carbohydrate signals for the Cyp 200 sample decreased less than those of the Cyp Ref sample. Namely, the impregnation of PEG 200 increased the ^13^C CP-MAS signals of carbohydrates at all temperature because the interaction between carbohydrates and PEG 200 enhanced the efficiency of ^1^H–^13^C magnetization transfer. In addition, the signals of PEG 200 overlapped with the carbohydrate signals that could be observed at 62 and 71 ppm only over 60 °C. The PEG 200 signals in the Cyp 200 sample were clearly observed in ^13^C PST-MAS NMR spectra as shown in Fig. 4B. These PEG 200 signals could be assigned as the terminal ethylene glycol unit (–O–CH_2_–*C̲*H_2_–OH: 64 ppm, –O–*C̲*H_2_–CH_2_–OH: 75 ppm) and inner ethylene glycol unit (–O–*C̲*H_2_–*C̲*H_2_–O–: 73 ppm). As far as the ^13^C PST-MAS NMR spectra for biomass constituents in Cyp 200, only OCH_3_ appeared as a weak signal and the remaining substituents of cypress could not be observed because of overlap with the signals of PEG 200. During cooling processes, the ^13^C PST-MAS signals of PEG 200 showed maximum intensities at 80 °C and decreased with subsequent cooling. This PST-MAS signal reduction could be explained by suppressed mobility of PEG molecules due to the temperature drop.

**Fig. 5:**
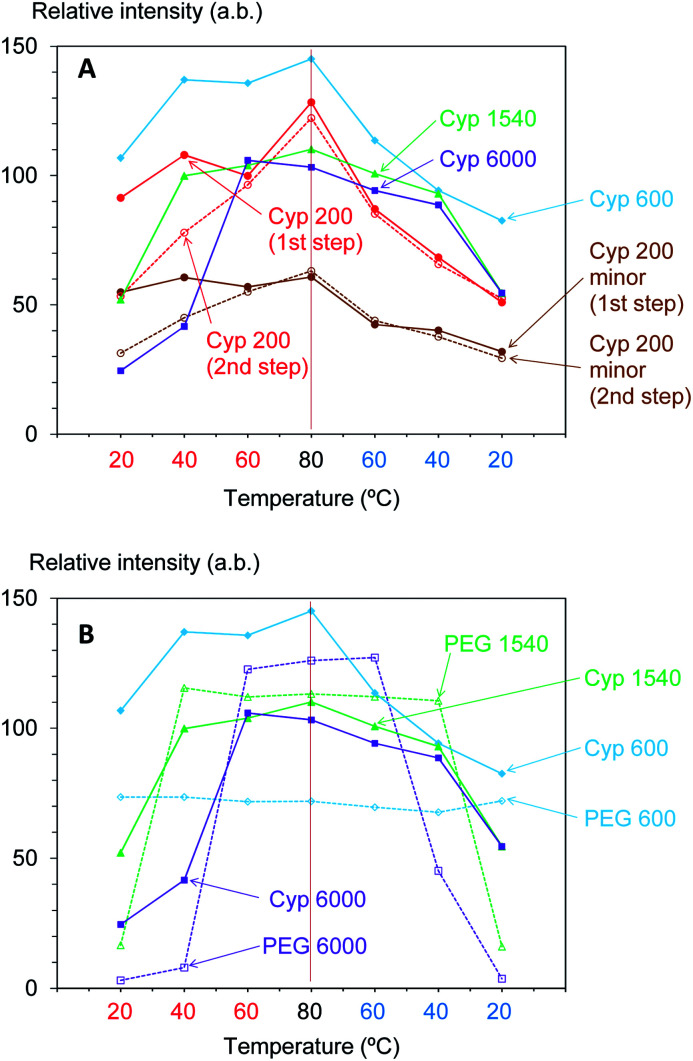
Change of ^13^C PST-MAS signal of PEG molecules: (A) in PEG-impregnated Japanese cypresses: (B) in pure form.

According to the above results, the ^13^C CP-MAS NMR method was suitable for analysing the biomass constituents in the impregnated cypress while the ^13^C PST-MAS NMR method was suitable for analysing PEG molecules in the impregnated cypress. In order to examine the effect of the molecular weight of PEG molecules on their behaviours in the impregnated cypress, variable temperature ^13^C PST-MAS NMR spectra were measured for the cypresses impregnated with various molecular weights of PEG molecules. The original ^13^C PST-MAS NMR spectra of PEG-impregnated cypresses are presented in Fig. S3 (ESI).† For each PEG-impregnated cypress, the signal intensity changes are summarized in Fig. 5A. As shown in Fig. S3,† the terminal glycol signals became smaller with increasing molecular weight of PEG. The intensity of the inner glycol signal at ambient temperature also decreased with increasing molecular weight of PEG. As shown in Fig. 5, the intensity of the inner glycol signal increased with increasing measuring temperature and decreased with decreasing temperature; however, the signal intensity curve for the temperature cycle was asymmetric. Concerning the PST-MAS signal of each PEG-impregnated cypress, the signals of Cyp 200 and Cyp 600 were significantly affected by the measuring temperature; consequently, they monotonously decreased during cooling. Meanwhile, the signals of Cyp 1540 and Cyp 6000 gradually decreased close below the melting point and then, they rapidly became smaller at 20 °C.

The PEG impregnation effect was more clearly understood by comparing PEG molecules in pure form with the impregnated cypress. The variable temperature ^13^C PST-MAS NMR spectra of selected PEGs (PEG 600, PEG 1540, and PEG 6000) are presented in Fig. S4 (ESI).† The comparison of ^13^C PST-MAS signal intensities between the impregnated cypresses and PEGs is summarized in Fig. 5B. For all PEGs, the ^13^C PST-MAS signals could be clearly observed above the melting temperature; their intensities were almost unchanged even at elevated temperatures. Therefore, the decrease of ^13^C PST-MAS signal on cooling was caused by the interactions between the PEG and biomass constituents in cypress. At the same time, the rate of ^13^C PST-MAS signal reduction was in proportional to the strength of the interaction between PEG and biomass constituents.

**Fig. 6:**
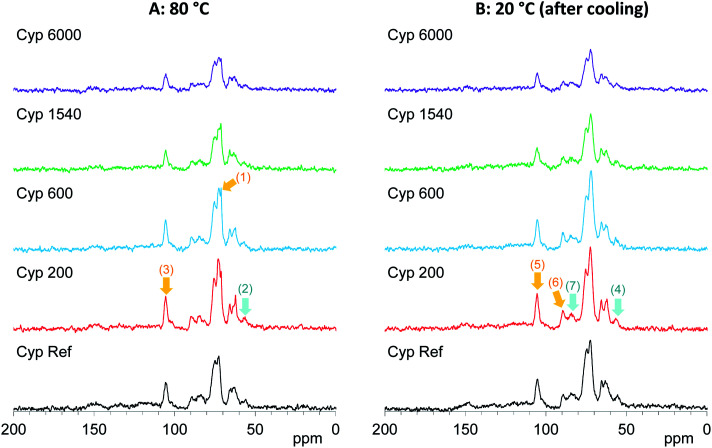
^13^C CP-MAS NMR spectral changes of Japanese cypress impregnated with various PEG molecules at 80 °C and 20 °C after cooling.

On the other hand, the ^13^C CP-MAS NMR method provided information about the effect of the PEG molecular weight on the behaviours of biomass constituents in the impregnated cypress. The original ^13^C CP-MAS NMR spectra of PEG-impregnated cypresses and PEG molecules in pure form are presented in Fig. S5 and S6, respectively (ESI).† As shown in Fig. 6, the ^13^C CP-MAS signal intensity changed depending on the molecular weight of the PEG. At 80 °C where all PEG molecules are present in liquid form (Fig. 6A), one can see the inner chain signal of PEG [71 ppm, arrow (1)]. The signal intensity of lignin OCH_3_ [57 ppm, arrow (2)] was not increased but the signal intensity of carbohydrates C1 [105 ppm, arrow (3)] was increased by the impregnation with shorter-chain PEG (Cyp 200, Cyp 600). In the ^13^C CP-MAS NMR spectra at 20 °C (Fig. 6B), although the intensity of ^13^C CP-MAS signals generally increased with cooling, the difference in the signal increase between lignin OCH_3_ [arrow (4)] and carbohydrates C1 [lignin (5)] still remained. At the same time, crystalline cellulose C4 [90 ppm, arrow (6)] increased more than amorphous cellulose C4 [84 ppm, arrow (7)]. The ^13^C CP-MAS signal increase due to the PEG impregnation was caused by the interaction between PEG molecules and biomass constituents in cypress; therefore, the shorter-chain PEG more interacted with carbohydrates, especially with crystalline cellulose, compared with lignin.

**Fig. 7:**
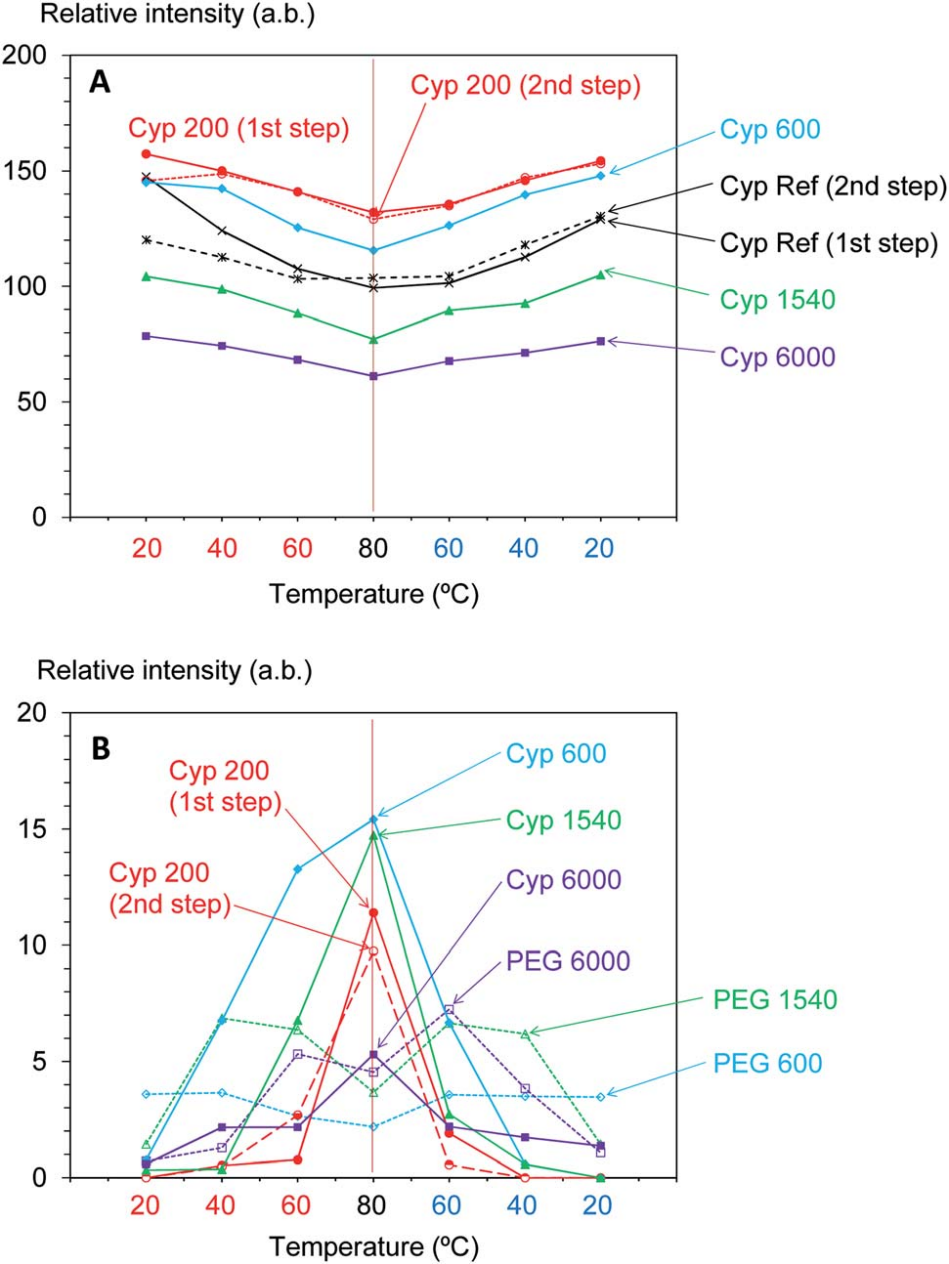
Change of ^13^C CP-MAS signal: (A) carbohydrates in cypresses: (B) PEG molecules in the cypress and pure form.

Furthermore, the intensity of the carbohydrate signals slightly decreased with warming and then increasing with cooling. Fig. 7A shows the intensity change of the maximum signal of carbohydrates (72 ppm), which were assigned as cellulose C2, 3, 5 signals, in the cycles of rising and dropping temperatures. As shown in Fig. S5,† the impregnation with PEG 600 also enlarged the signal intensity of cellulose; however, the growth rate of the Cyp 600 signal was smaller than that of Cyp 200. In contrast, the impregnation of even longer-chain PEG (PEG 1540, PEG 6000) reduced the signal intensity of cellulose. That is, the signal intensity decreased in the following order: Cyp 200 > Cyp 600 > Cpy 1540 > Cyp 6000. The signal decrease caused by long chain PEG molecules resulted from the reduction of ^1^H–^13^C CP efficiency due to the interaction between carbohydrates and PEG molecules.

As the inner chain signal of PEG (71 ppm) in all PEG-impregnated cypresses could be observed at 80 °C (Fig. 6A), this PEG signal was monitored during the temperature cycles. The signal changes of the PEG's inner chain (71 ppm) in the ^13^C CP-MAS NMR spectra are summarized in Fig. 7B. Although the CP-MAS signal of PEG in the impregnated cypress could not be observed at ambient temperature, the signal enlarged at the lower measurement temperatures with the impregnation of short chain length PEG. The CP-MAS signal of PEG molecules in Cyp 200 showed a different trend, as in this case PEG 200 had three signals which overlapped with the carbohydrate signals. As shown in Fig. S6 (ESI),† the signal of PEG in solid form (PEG 1540, PEG 6000) showed a relatively broad CP-MAS signal for the woody products, while the signal of PEG in liquid form (PEG 600) showed a sharp PST-MAS signal. Although all CP-MAS signals of PEG molecules in pure form became sharp at higher measurement temperatures, the signal intensity decreased with increasing temperature, as shown Fig. 7B. The different trend of signal intensity changes between PEG molecules in cypress and in pure form was also caused by the interaction between PEG molecules and carbohydrates in the impregnated cypress. According to the results of CP-MAS NMR spectra, the interaction of short chain length PEG molecules was larger than that of long chains.

### Variable temperature *T*_1_H changes of PEG-impregnated Japanese cypresses

**Fig. 8:**
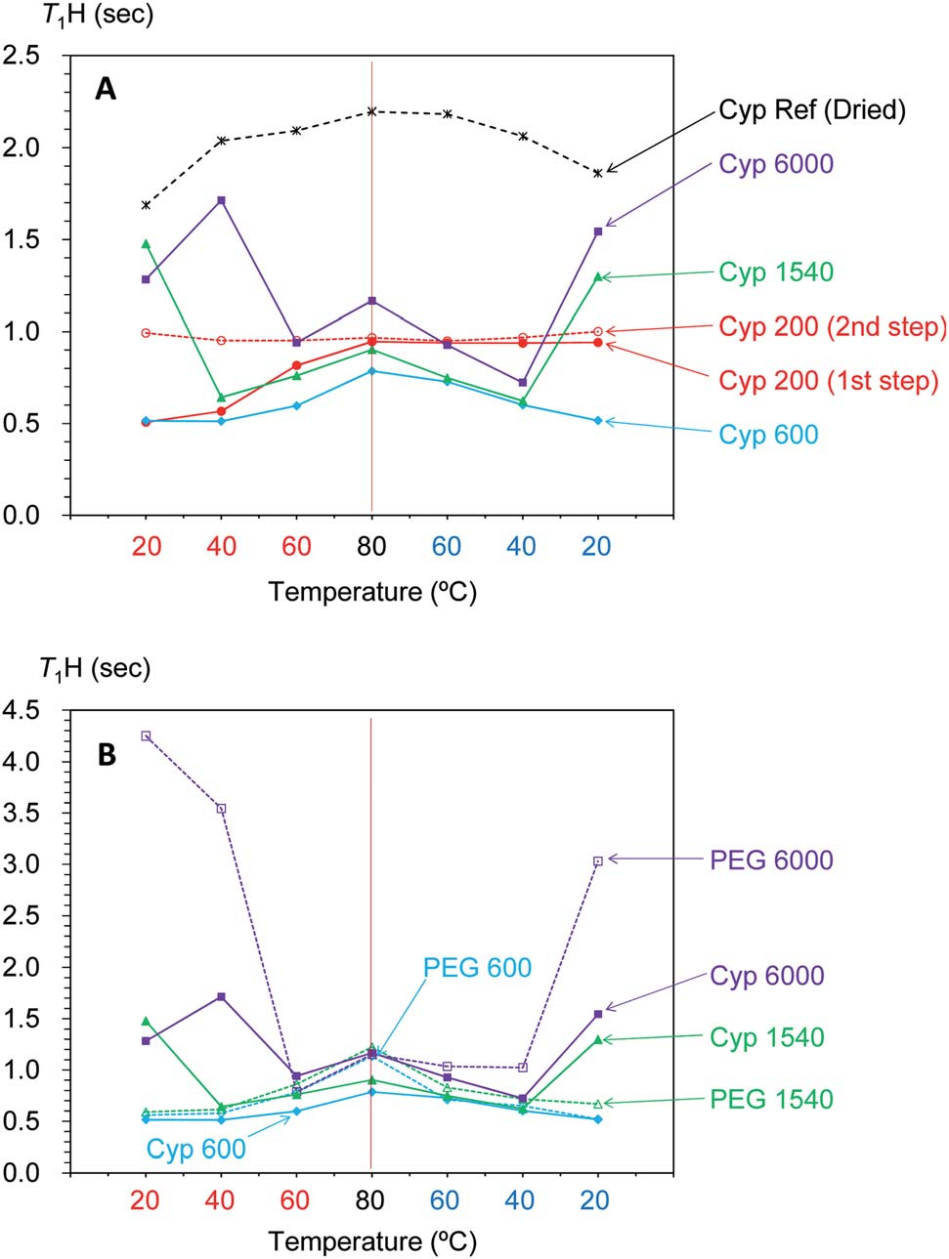
Change of *T*_1_H value of PEG molecules: (A) in PEG-impregnated Japanese cypresses: (B) in pure form.

In our previous studies, variable temperature ^1^H spin-lattice relaxation times in the laboratory frame (*T*_1_H) during temperature cycles have provided information about changes of molecular mobility in Japanese cypress due to the isolation of biomass constituents^23^ and resin impregnation.^26^ In order to evaluate the interactions of PEG molecules and biomass constituents in the PEG-impregnated Japanese cypress with ^1^H spin diffusion, *T*_1_H values of PEG-impregnated Japanese cypresses were examined by variable temperature measurements (Fig. 8).

Similar to the intensity of the ^1^H signal, the *T*_1_H value of PEG-impregnated cypress significantly changed around the melting point of the corresponding PEG in pure form (Fig. 8A). For the PEGs having a melting point lower than ambient temperature, the *T*_1_H value either stayed almost unchanged with temperature (Cyp 200), or increased with increasing temperature (Cyp 600). Meanwhile, for the PEGs having a melting point higher than ambient temperature, the *T*_1_H minimized value around the melting point. Similar to the ^1^H signal curve *versus* temperature, Cyp 1540 showed a similar *T*_1_H-temperature curve in both legs of the cycle, while Cyp 6000 showed a minimum *T*_1_H value at a lower temperature when being cooled. In the other words, PEG 6000 existed as a liquid phase in the cypress even below the melting point during cooling. The asymmetrical intensity–temperature curves of both ^1^H and ^13^C PST-MAS signals of Cyp 6000 were also related to this lowered melting point of PEG 6000.

The ^1^H spin-lattice relaxation time in the laboratory frame (*T*_1_H) shows a V-shaped curve against temperature and the minimum value appeared at the temperature where the rotational correlation time (*τ*_c_) matches the Larmor frequency (*τ*_c_*ω*_0_ = 1; *ω*_0_ is the Larmor frequency). Therefore, solid-state PEG had a longer *T*_1_H value that reflected a larger correlation time *τ*_c_ (*τ*_c_*ω*_0_ > 1). In contrast, liquid-state PEG has a shorter correlation time *τ*_c_ (*τ*_c_*ω*_0_ < 1) and the *T*_1_H value increased with decreasing *τ*_c_ (increasing temperature). Actually, the *T*_1_H values of PEG molecules (except PEG 200) in the cypress increased with increasing temperature in the liquid state because the mobility of PEG molecules increased with increasing temperature.

Next, by comparing the *T*_1_H values of the PEG-impregnated cypress with the corresponding PEG in pure form, impregnation effects on the *T*_1_H value were examined by variable temperature measurements (Fig. 8B). For PEG 600 and PEG 1540, which had medium chain lengths, the *T*_1_H value of the PEG molecules increased with increasing temperature in the pure form as well as in the PEG-impregnated cypress (except Cyp 1540 at 20 °C). However, the rate of the *T*_1_H increase was faster and the *T*_1_H value at 80 °C for PEG 600 and PEG 1540 in pure form was long compared with that in the PEG-impregnated cypress. Therefore, the suppression of the *T*_1_H increase in Cyp 600 and Cyp 1540 (lowered *T*_1_H value at 80 °C) was caused by the ^1^H spin diffusion due to the interaction between liquid phase PEG molecules and biomass constituents in the cypress. In contrast, the long-chain PEG 6000 length *T*_1_H values remained similar, even at 80 °C, indicating that the ^1^H spin diffusion of liquid phase PEG 6000 in the pure form was unchanged by the impregnation into cypress. Meanwhile, solid phase PEG 6000 in pure form showed considerably longer *T*_1_H values at ambient temperature, which was reduced by the impregnation into cypress, as shown by the shorter *T*_1_H value of Cyp 6000. These reductions of *T*_1_H value due to the impregnation were caused by the interaction between PEG molecules and the biomass constituents in the cypress. The interaction was higher at high temperatures in liquid phase PEG molecules for Cyp 600 and Cyp 1540 but was higher at low temperatures in solid phase PEG molecules for Cyp 6000.

### Infrared spectroscopy of the PEG impregnated Japanese cypresses

**Fig. 9:**
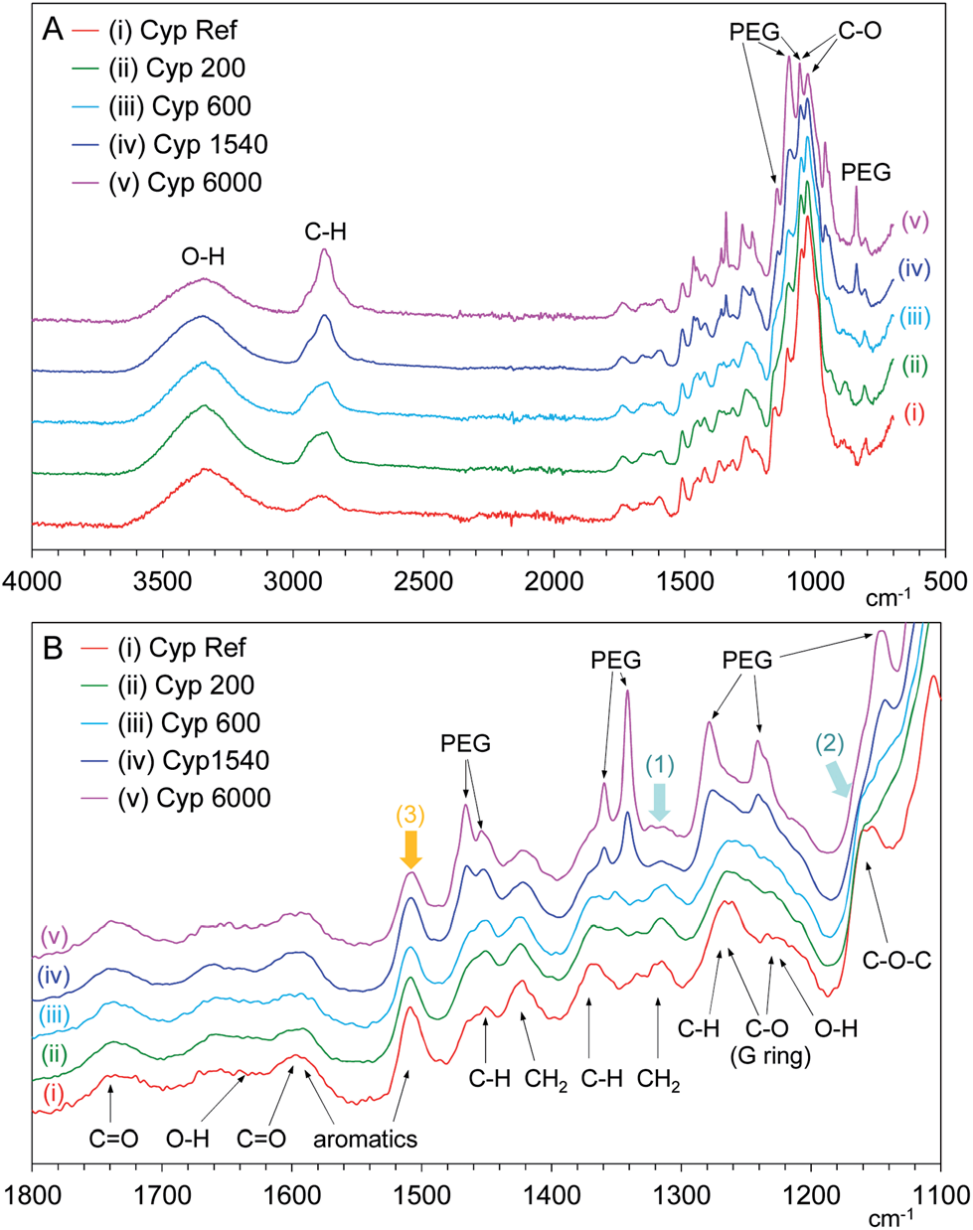
ATR-IR spectra of Japanese cypress with various molecular weight PEG molecules.

FT-IR spectroscopy is powerful tool for analysing changes of functional groups in materials. In particular, the ATR method is suitable for surface observation to provide significant information on the lumen of cell walls in coniferous wood. Fig. 9 shows changes in the normalized ATR-IR spectra of PEG impregnated Japanese cypresses: the full range spectrum is shown in Fig. 9A, and a magnified view between 1000–1800 cm^−1^ is shown in Fig. 9B. Assignments of characteristic vibration bands for Japanese cypress were based on our previous work on Japanese cypress^23^ as well as a review about ATR-IR spectra of wood and cellulose.^29^

In the full range spectra (Fig. 9A), one can see significant cypress bands at 3100–3500 cm^−1^ (O–H stretch), 2800–2950 cm^−1^ (C–H stretch), and 980–1080 cm^−1^ (C–O bond vibrations). Newly appeared bands with the impregnation of PEG were observed near 840, 1060, 1100, and 1150 cm^−1^ in the low wave number region (C–O bond vibrations) and at 2800–2950 cm^−1^ overlapped with cypress (C–H stretch). In the magnified view (Fig. 9B), the PEG vibration bands appeared anew at 1220–1300 cm^−1^ (C–O stretch), 1330–1370 cm^−1^ (C–H bending), 1440–1480 cm^−1^ (C–H bending) with the impregnation. Interestingly, the intensities of PEG vibration bands increased with increasing the molecular weight of PEG impregnated. Namely, the band intensities of short-chain PEG molecules (PEG 200, 600) were lower, even though the intensities of ^1^H MAS and ^13^C PST-MAS signals of short-chain PEG molecules were rather larger than those of long-chain PEG molecules (PEG 1540, 6000). As mentioned above, solid-state NMR can be used for the bulk analysis for woody materials while ATR-IR is restricted to surface analysis for woody materials. Therefore, the ATR-IR spectra also showed that the longer-chain PEG molecules existed on the lumen of impregnated cypress because the longer-chain PEG molecules could not infiltrate into the intercellular region of the cell wall.

Furthermore, the ATR-IR spectra of PEG-impregnated cypresses gave information about the interaction between PEG molecules and biomass constituents at the lumen. With increasing the chain length of PEG molecules, the CH_2_ bending [1300–1360 cm^−1^, arrow (1)] and C–O–C vibration [1125–1180 cm^−1^, arrow (2)] that originated from carbohydrates were reduced while the aromatic band of lignin [1495–1520 cm^−1^, arrow (3)] remained almost unchanged, as shown in Fig. 9B. On the surface of lumen, the longer-chain PEG molecules preferably covered over carbohydrates, compared with lignin. Combining this data with the results of ^13^C CP-MAS NMR in the shorter-chain PEG molecules, all PEG molecules predominately interacted with carbohydrate rather than lignin, regardless of chain length.

### Nanostructure of the PEG impregnated Japanese cypresses

**Fig. 10:**
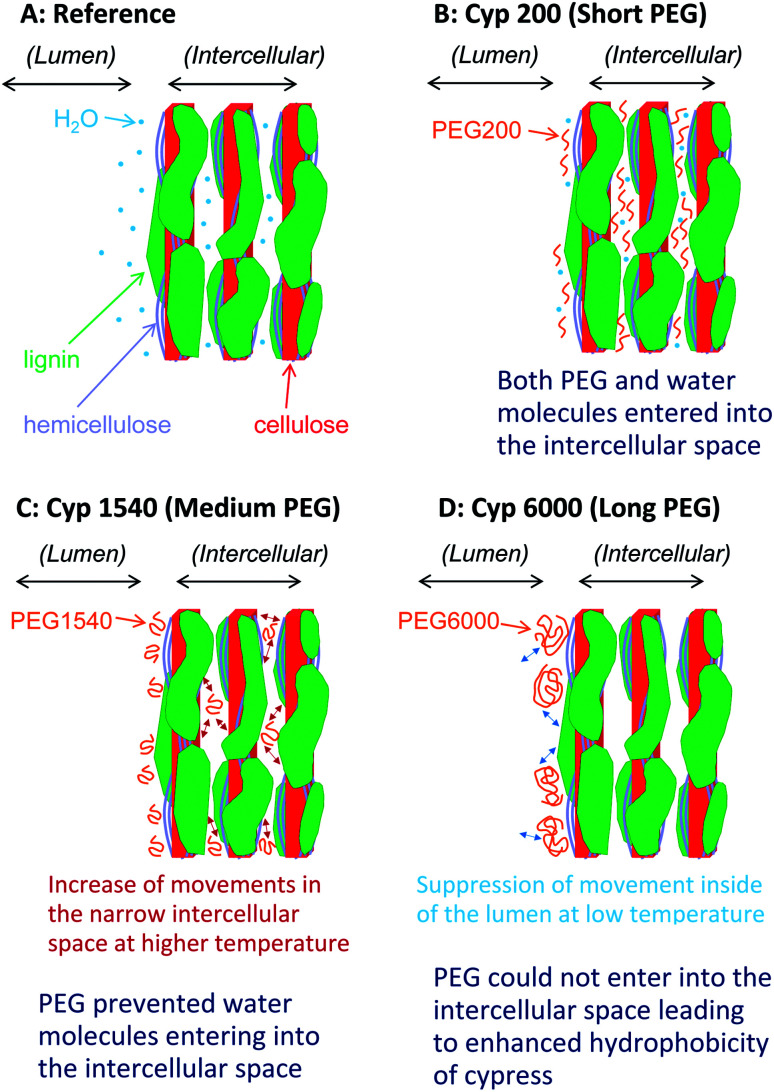
Nanostructures of Japanese cypress impregnated with different chain length PEG molecules after storage under controlled moisture conditions in a desiccator.

Based on results on plant materials in previous publications^30,31^ and our previous studies about chemical modifications,^23,32^ we have proposed a nanostructural model of the PEG-impregnated cypress, which has the hierarchical structure formed by its biomass constituents (cellulose, hemicellulose, and lignin) shown in Fig. 10. The sapwood of Japanese cypress has a repeating structure of lumen surrounded by cell wall. A cellulose microfibril is the basic biomass constituent maintaining the shape of the cell wall. The cellulose microfibril connects with hemicellulose to form a cellulose–hemicellulose aggregate. A three-dimensional network of lignin covers the cellulose–hemicellulose aggregate through a lignin carbohydrate complex (LCC). The cell wall consists of this hierarchical structure including the carbohydrates and lignin, which makes intercellular nanopores inside and micro-scale lumens outside the cell.

Before the impregnation with PEG [Fig. 10A (Cyp Ref)], both lumen and intercellular nanopore in the cell wall held a considerable amount of water molecules because of the easy accessibility of water molecules into the intercellular space. Hence, the Cyp Ref sample easily released molecular water with increasing temperature. The release of water molecules appeared not only as a reduction of the water signal in the ^1^H MAS NMR spectra directly but also indirectly as a slight reduction of the carbohydrate signals in ^13^C CP-MAS NMR spectra.

During impregnation, the short ethylene glycol chain PEG 200 [Fig. 10B (Cyp 200)] also easily accessed the intercellular space as did water molecules. Even in the temperature cycles, Cyp 200 released water molecules but it kept PEG 200 molecules in the nanopores of the intercellular space. Since PEG 200 molecules could interact with the biomass constituents instead of solely with themselves, the spin–spin relaxation of PEG 200 was suppressed at higher temperatures, boosting the rate of signal increase due to the temperature rise in both ^1^H and ^13^C PST-MAS NMR spectra. The interaction of PEG 200 molecules with carbohydrates, especially with crystalline cellulose, in the nanopores of the intercellular space enhanced the ^1^H–^13^C magnetization transfer to increase the intensity of the carbohydrate signals in ^13^C CP-MAS NMR spectra.

The impregnation of PEG 1540, which has a chain length seven times that of PEG 200, was more abundant over the lumen than the intercellular space of cell wall. The PEG 1540 molecules prevented access of water molecules into the intercellular space because of steric hindrance of PEG molecules over the cell wall [Fig. 10C (Cyp 600)]. Actually, the higher content of PEG 1540 over the lumen increased the intensity of ATR-IR bands of PEG molecules. Moreover, the impregnation of longer-chain PEG brought the reduction of carbohydrate bands in ATR-IR spectra, indicating that PEG molecules interacted with carbohydrates to prevent the infiltration of water molecules due to the increased bulk *via* hydroxyl groups of carbohydrates. Since the suppression of spin–spin relaxation with increasing temperature was moderate because of the higher viscosity of PEG 1540, the rate of signal increase in ^1^H and ^13^C PST-MAS NMR spectra was smaller than for PEG 200. Furthermore, the lower interaction between PEG 1540 molecules and the biomass constituents in Cyp 1540 suppressed the ^1^H–^13^C magnetization transfer to decrease the intensity of the carbohydrate signals in the ^13^C CP-MAS NMR spectra.

With the impregnation of PEG 6000 having the longest chain length [Fig. 10D (Cyp 6000)], the infiltration of both water and PEG 6000 molecules into the intercellular space were prevented. The impregnation occurred over the surface of the cell wall contacting the lumen, resulting in signal intensity reductions of both carbohydrates and PEG 6000 in the ^13^C CP-MAS NMR spectra of Cyp 6000. At the same time, the intensity of ATR-IR bands of PEG 6000 in Cyp 6000 increased more than for other PEG molecules. Furthermore, ^1^H spin-lattice relaxation at high temperatures was suppressed because the impregnation of PEG 6000 occurred only at the lumen-cell wall interface. Thereby, both Cyp 6000 and PEG 6000 in pure form had similar *T*_1_H values, even though Cyp 1540 has a shorter *T*_1_H value than PEG 1540 in the pure form because of the inclusion of PEG molecules in the liquid phase inside nanopores, which enhanced ^1^H spin-lattice relaxation at high temperatures due to the degree of movements in the narrow space (Fig. 10C). In contrast, PEG 6000 in pure form had a significantly longer *T*_1_H value than Cyp 6000 at ambient temperature, indicating that PEG 6000 in the solid phase interacted with the lumen of impregnated cypress to enhance ^1^H spin-lattice relaxation because the suppression of movement inside of the lumen, resulting in the increase of the contact area between PEG molecules and biomass constituents (Fig. 10D).

As shown above, the variable temperature measurements of solid-state NMR spectra and relaxation times were useful for evaluating not only the hydrophobicity but also the distribution of chemicals in the lumen and the nanopores in the intercellular space. These measurements are also an effective way to investigate the interaction between other chemicals, which have various molecular sizes and is a way to investigate molecular mobility, and the nature of the biomass constituents in woody materials. In the future, we are planning to investigate the behaviour and interactions of various chemicals in wood composites from the point of view of the utilization of woody composites with well-controlled manufacturing qualities.

## Conclusions

The effects of the chain lengths of polyethylene glycol (PEG) impregnated into coniferous wood (Japanese cypress) were studied by variable temperature solid-state NMR spectra and nuclear magnetic relaxation times. The ^1^H MAS NMR spectra at ambient temperature showed that the hydrophobicity of the impregnated Japanese cypress increased with increasing the chain length of PEG. In a cycle of rising and dropping temperatures, water molecules were easily released at temperatures over 60 °C but all PEG molecules remained in the cypress. The variable temperature ^13^C PST-MAS NMR spectra of the PEG molecules showed similar trends in signal intensity to the ^1^H MAS NMR spectra. The ^13^C CP-MAS signal of carbohydrates in the PEG-impregnated cypress slightly decreased with increasing measurement temperature; however, the signal intensity of carbohydrates decreased proportionally to the chain length of the PEG. Meanwhile, the ^13^C CP-MAS signal of PEG molecules in the PEG-impregnated cypress increased with increasing temperature. At the same time, the long chain PEG molecules (PEG 6000) showed weak CP-MAS signals while other PEG molecules provided larger signals than in the pure form. For medium chain length PEG (PEG 600, 1540) in the liquid phase, *T*_1_H values showed a maximum value at 80 °C, although long chain length PEG (PEG 6000) in the solid phase had a considerable larger *T*_1_H value at ambient temperature. The variable temperature solid-state NMR spectra and relaxation times indicated that the short chain PEG molecules penetrated into the nanopores in the intercellular space of the cell wall together with water molecules. With a longer chain length, PEG molecules could not access the nanopores as indicated by different trends in the solid-state NMR and relaxation times. These distributions of PEG molecules were also supported by ATR-IR spectra, which more PEG molecules attached to the lumen of cell wall. Both ^13^C CP-MAS NMR and ATR-IR spectra showed that PEG molecules mainly interacted with carbohydrates. Therefore, the increase of hydrophobicity was caused by the increase of bulk effect of PEG molecules attached to carbohydrates, especially in the lumen of the cell wall. This type of variable temperature measurements will be applied to woody composites having constituents with different molecular sizes and mobilities to provide information about the distribution of chemicals as well as interaction between chemicals and biomass constituents in the woody composites.

## Conflicts of interest

There are no conflicts to declare.

## Supplementary Material

RA-009-C9RA01848D-s001
