# Supplementary material for: Variable temperature solid-state NMR spectral and relaxation analyses of the impregnation of polyethylene glycol (PEG) into coniferous wood
Source: RSC Adv. 2019 May 20;9(27):15657–67. doi: 10.1039/c9ra01848d (PMC9064324; doi:10.1039/c9ra01848d)
Supplement: RA-009-C9RA01848D-s001 [file RA-009-C9RA01848D-s001.pdf]

Electronic Supplementary Material (ESI) for RSC Advances.
This journal is © The Royal Society of Chemistry 2019

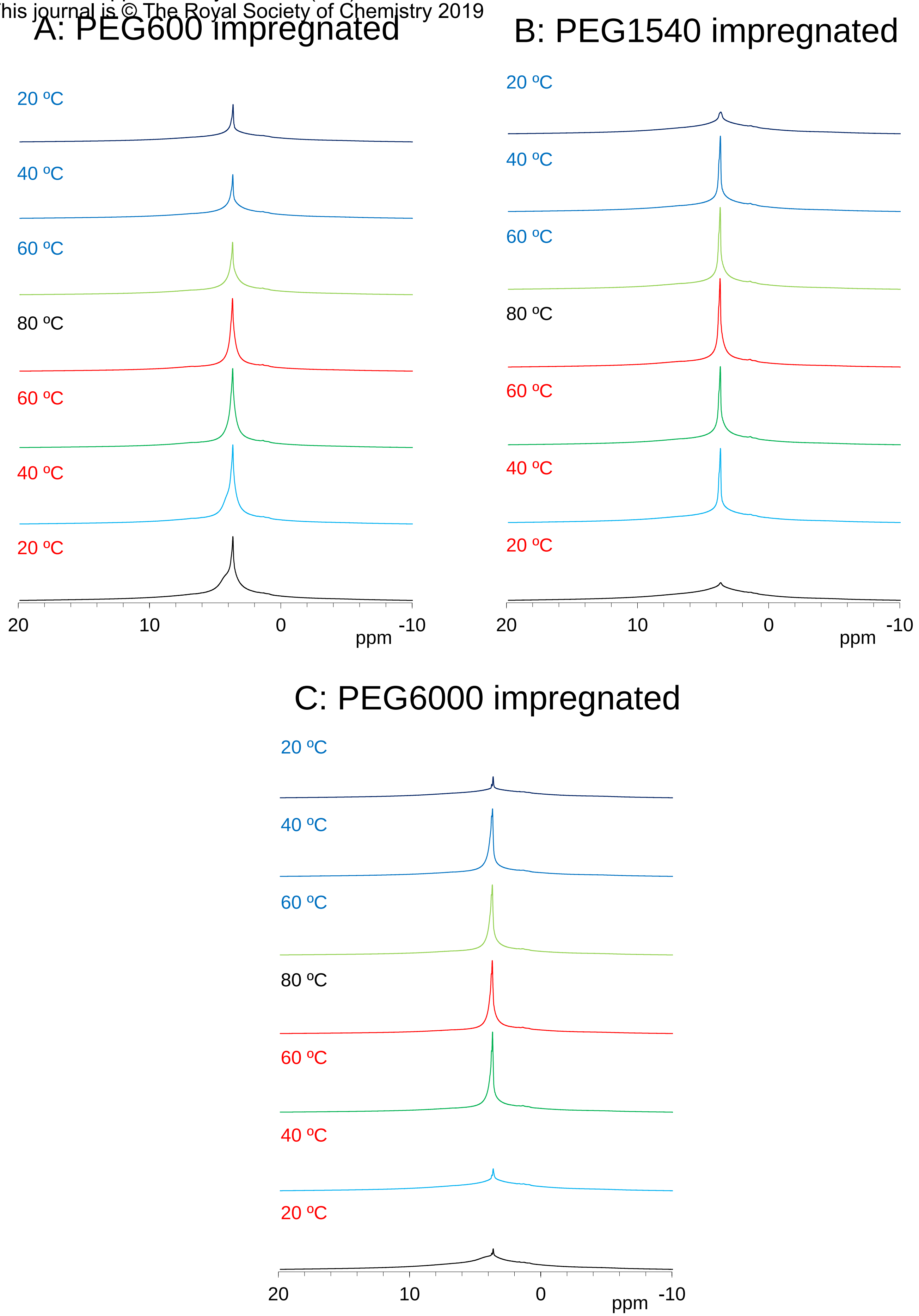

Figure S1. Variable temperature ^{1}H MAS NMR spectra of PEG impregnated Japanese cypresses

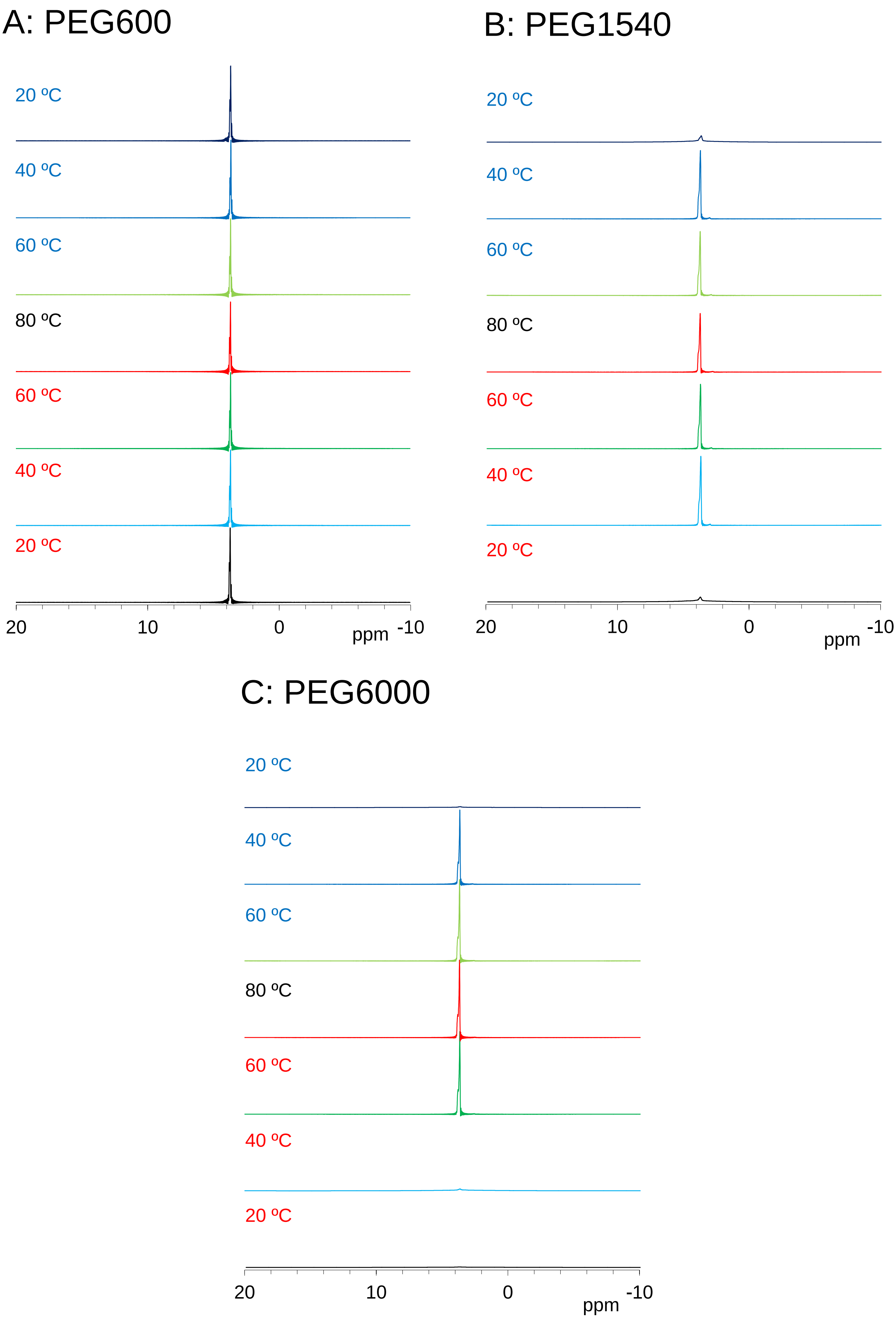

Figure S2. Variable temperature ^{1}H MAS NMR spectra of PEG

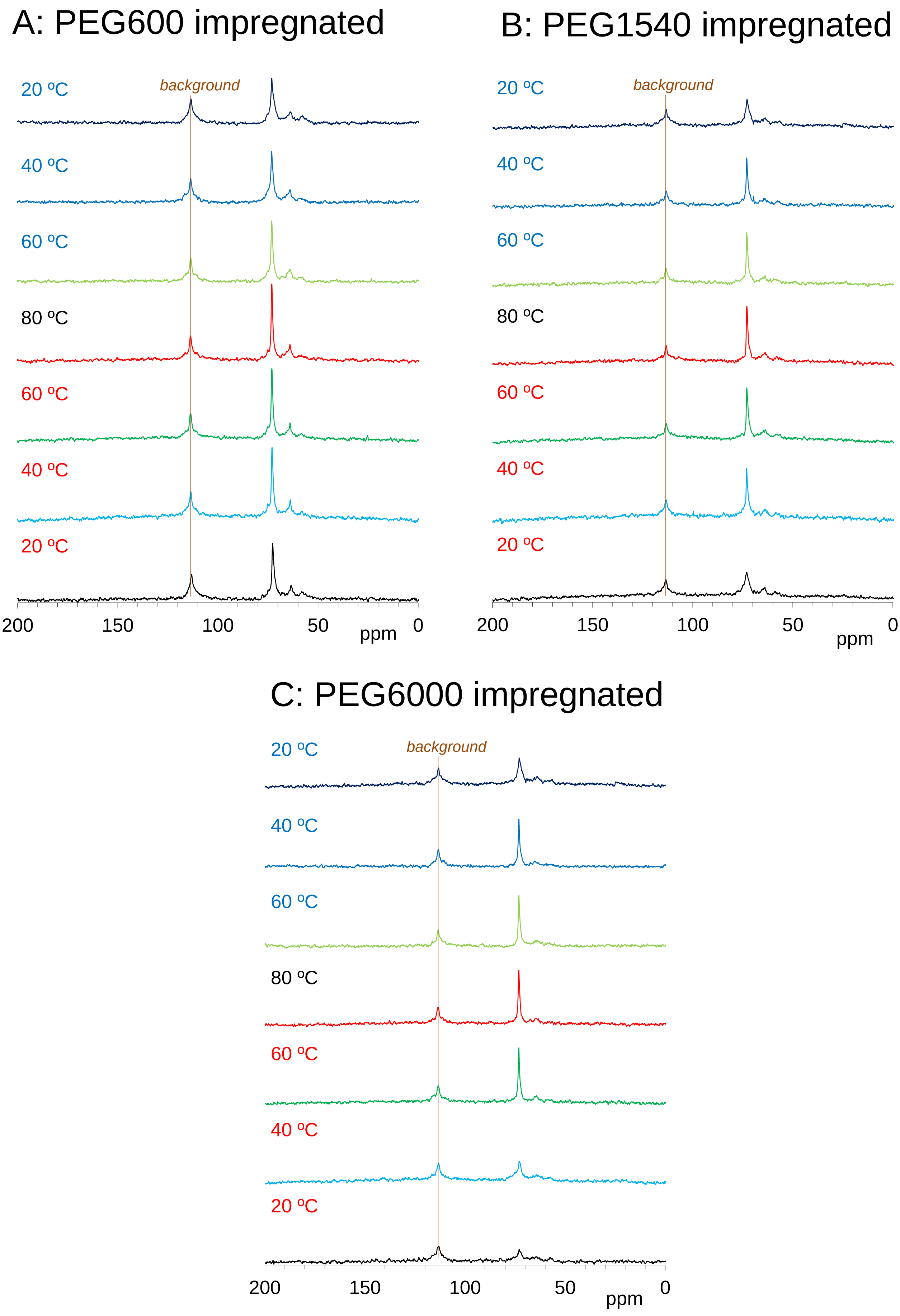

Figure S3. Variable temperature ^{13}C PST-MAS NMR spectra of PEG impregnated Japanese cypresses

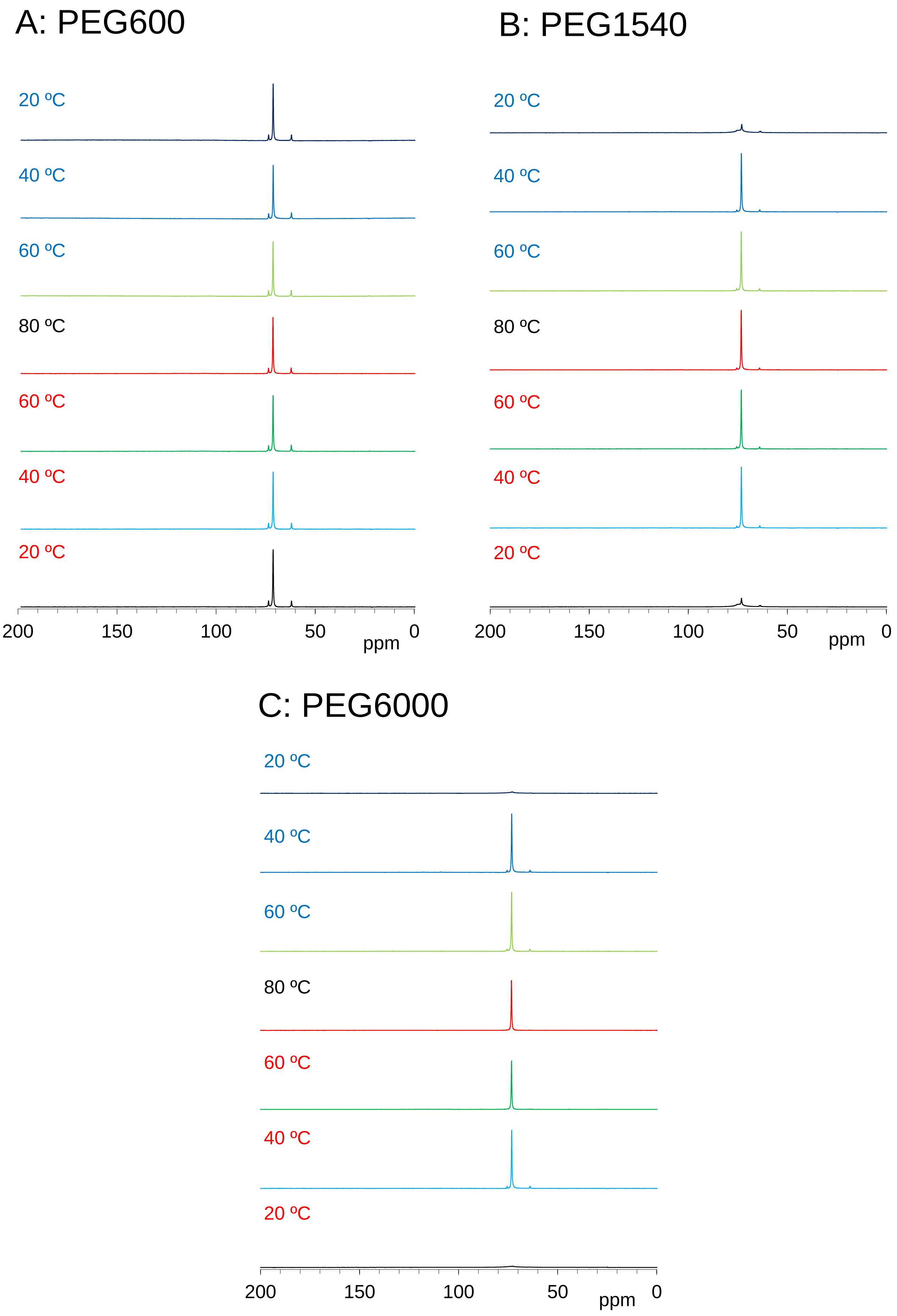

Figure S4. Variable temperature ^{13}C PST-MAS NMR spectra of PEG

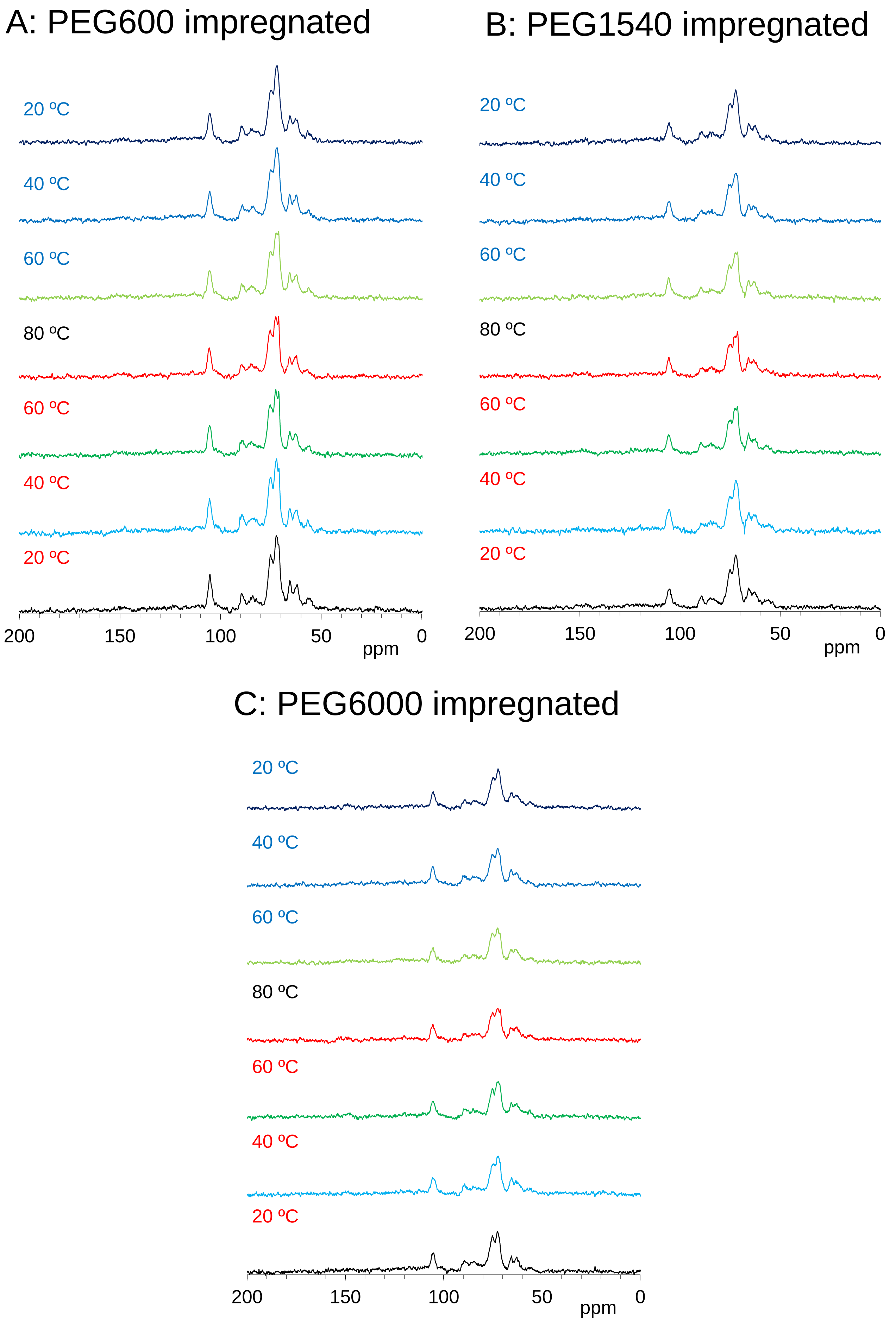

Figure S5. Variable temperature ^{13}C CP-MAS NMR spectra of PEG impregnated Japanese cypresses

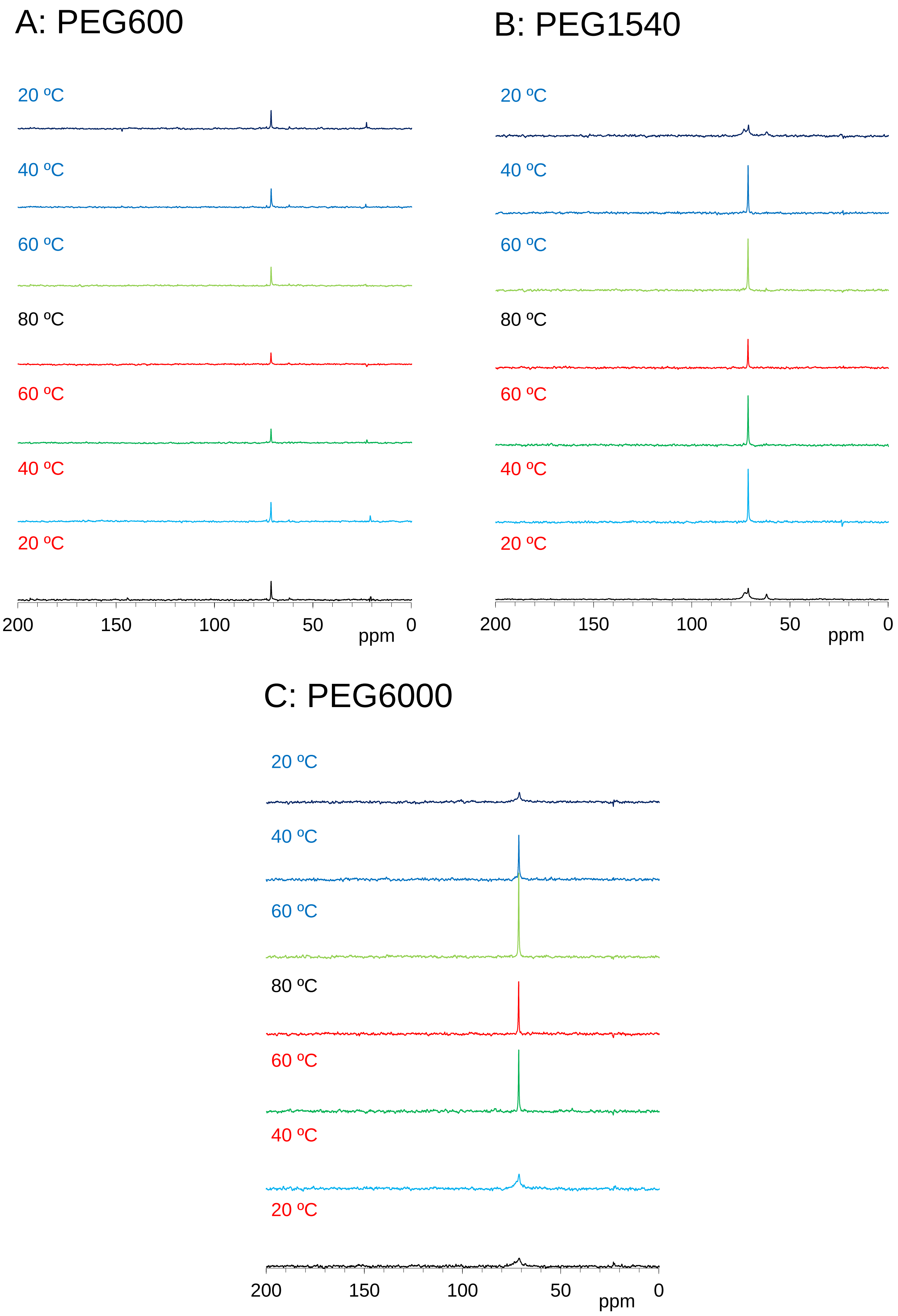

Figure S6. Variable temperature ^{13}C CP-MAS NMR spectra of PEG
